# A Dynamic Thermal Camouflage Metadevice with Microwave Scattering Reduction

**DOI:** 10.1002/advs.202201054

**Published:** 2022-06-05

**Authors:** Liming Yuan, Cheng Huang, Jianming Liao, Chen Ji, Jingkai Huang, Yuetang Wang, Xiangang Luo

**Affiliations:** ^1^ State Key Laboratory of Optical Technologies on Nano‐Fabrication and Micro‐Engineering Institute of Optics and Electronics Chinese Academy of Sciences Chengdu 610209 China; ^2^ School of Optoelectronics University of Chinese Academy of Sciences Beijing 100049 China

**Keywords:** dynamic thermal camouflage, metadevice, microwave scattering reduction, microwave‐infrared compatibility

## Abstract

With rapid development of radar and infrared (IR) surveillance technologies, the need for microwave‐IR compatible camouflage is now more than ever. Here, a novel multispectral metadevice is proposed to simultaneously achieve microwave scattering reduction, dynamic IR camouflage, and low IR reflection. This metadevice is constructed by the coding thermoelectric elements with the properly designed phase arrangement, and the incident microwave energy can be redirected to the nonthreatening directions for specular reflection reduction. The dynamic IR camouflage with low IR reflection is realized by using the thermoelectric cooling and heating effect and high‐IR‐absorptivity surface. The above three functionalities are demonstrated by experimental measurement. The 10 dB scattering reduction can be realized at the microwave band of 10–16.1 GHz. In the IR region, the designed metadevice can not only dynamically modulate the surface temperature for matching different background temperatures, but also realize the pixel temperature control for adapting to a spatially varying thermal background. In addition, it reflects almost no surrounding thermal signals compared with the traditional low‐emissivity IR stealth material. This study paves an effective way to achieve microwave‐IR compatible camouflage, which may inspire the future researches and applications in multispectral camouflage and stealth fields.

## Graphical abstract

This report presents an innovative thermoelectric metadevice, which can not only reduce the microwave specular scattering by beam‐splitting effect, but also achieve dynamic IR camouflage by the modulation of surface temperature along with minimizing the reflection of surrounding thermal signals. The multifunctional metadevice paves an effective way to researches of multispectral camouflage and stealth fields.

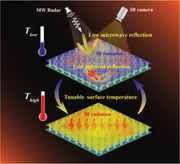

## 1 Introduction

Over millions of years of evolution, camouflage is extensively adopted by various species such as *cephalopods* and *chameleons* to escape from predators against extinction.^[^ ^1^, ^2^ ^]^ Nowadays, camouflage technology has played more and more important role in modern military confrontation. It can effectively render military targets invisible from potential threats by concealing signals such as microwave, IR, laser, visible, etc. Owing to explosive developments and applications of radar and thermal surveillance technologies,^[^ ^3^, ^4^ ^]^ microwave‐ IR compatible camouflage has garnered much attention. The conventional strategy is to cover a microwave absorber with an IR functional layer,^[^ ^5^, ^6^, ^7^, ^8^, ^9^, ^10^, ^11^, ^12^, ^13^, ^14^ ^]^ such as ultra‐thin IR coating,^[^ ^5^, ^6^, ^7^ ^]^ metallic frequency selective surface,^[^ ^8^, ^9^, ^10^, ^11^, ^12^ ^]^ 1D all‐dielectric photonic structure,^[^ ^13^, ^14^ ^]^ etc. The core of the conventional strategy is dominated by the IR functional layer, which is designed to satisfy the requirement of low IR emissivity and high microwave transparency. In some sense, the above reported achievements have provided effective ways for the microwave‐IR compatible camouflage. However, there are some inevitable drawbacks associated with this kind of IR functional layer, e.g., low IR emissivity also means high reflectance, resulting in intense reflection of thermal signals from the surrounding, easy contamination by dust or moisture, and limited IR camouflage effect in some scenarios (e.g., in deserts) where the target temperature is lower than the surrounding. Furthermore, there are many difficulties to integrate an IR functional structure with dynamically tunable surface emissivity into a microwave functional structure, because additional functional modules should be equipped for loading external stimuli, e.g., the electrode for electrical stimuli.^[^ ^15^, ^16^, ^17^ ^]^ These essential modules always have a great influence on microwave transmission, leading to the incompatibility between the microwave and IR functional structures. In addition, the researches on dynamic control of surface emissivity have also encountered other problems such as low tunability,^[^ ^18^ ^]^ narrow spectral window^[^ ^19^ ^]^ and nonreversible control,^[^ ^20^ ^]^ hindering the realization of adaptive thermal camouflage materials.

Actually, there are two approaches adopted to realize IR camouflage according to the Stefan–Boltzmann law, i.e., the emissivity manipulation approach on the one hand and the temperature manipulation approach on the other hand. Usually, the emissivity manipulation approach is to explore low‐emissivity (low‐e) coatings for shielding the thermal signal of an target,^[^ ^9^, ^13^, ^21^, ^22^, ^23^, ^24^ ^]^ while the temperature manipulation approach aims to modulate the surface temperature.^[^ ^25^, ^26^, ^27^, ^28^ ^]^ The both approaches are further extended to dynamically tunable camouflage to match the thermal signal with the background.^[^ ^15^, ^16^, ^17^, ^20^, ^27^, ^28^, ^29^ ^]^ Up to now, most efforts have been poured into the emissivity manipulation approach, while the temperature manipulation approach is just explored at the fundamental level. Because of the aforementioned drawbacks of the emissivity manipulation approach, the recent attention has been turned to the temperature manipulation approach for directly manipulating the surface temperature, such as utilizing a thermoelectric device (TED). Based on the thermoelectric cooling and heating effect, a flexible TED was proposed to realize the IR camouflage effect within a wide range of background temperature.^[^ ^27^ ^]^ Besides, a multispectral camouflage device was developed by integrating a thermochromic layer on the outer surface of the TED,^[^ ^28^ ^]^ which can achieve the instantaneous cloaking effect in both visible and IR regions. As the TED realizes IR camouflage by manipulating the surface temperature, it is independent of the surface emissivity, and then an outer surface with high IR emissivity (i.e., high IR absorptivity) could be allowed to minimize the reflectance of the surrounding thermal signal. On the base of the TED, the microwave‐IR compatible strategy would be also transferred to explore a microwave functional structure rather than the IR functional layer. Although the straightforward approach is to cover a TED with a microwave functional coating, this crude design leads to some imperfections such as huge bulk and heavy mass, which are unfavorable for practical applications. Fortunately, the emergence of coding metamaterial may provide the novel route to address the challenge.^[^ ^30^, ^31^, ^32^, ^33^ ^]^ It is generally composed of subwavelength elements with distinct phase responses, and the reflected wave can be modulated by suitably tailoring the phase of each element. Based on this principle, a plenty of novel low‐scattering materials and devices have been developed in microwave region.^[^ ^34^, ^35^, ^36^, ^37^, ^38^, ^39^, ^40^, ^41^ ^]^ They redirect the backscattering waves to other nonthreatening directions, working on the same principle with the geometry shaping technique that has been widely adopted in the stealthy design of advanced fighter and warship. Here, we introduce the concept of coding metamaterial into the TED design for constructing a novel thermoelectric metadevice (TEMD). By virtual of properly designed TE pillars and their electrode structure, the TEMD element can perform as a geometrical phase cell to modulate the reflection phase, and then through special arrangement of each element in a space‐variant manner, the whole TEMD can further achieve microwave scattering reduction. In addition, its intrinsic capability of active cooling and heating is still reserved for surface temperature manipulation. Both simulation and measurement results have demonstrated that the 10 dB specular scattering reduction is realized over a wide band ranging from 10 to 16.1 GHz, and the IR thermal camouflage is achieved by dynamically tuning the surface temperature to match the ambient temperature. In addition, our TEMD can also control the pixel temperature for adapting to a spatially varying thermal background. To the best of our knowledge, it is the first time for the TED‐based metadevice to simultaneously achieve microwave scattering reduction and dynamic IR camouflage. It is believed that our design paves an effective way to explore this kind of advanced camouflage technology.

## 2 Result and Discussion

### 2.1 Design of TEMD

**Figure 1:**
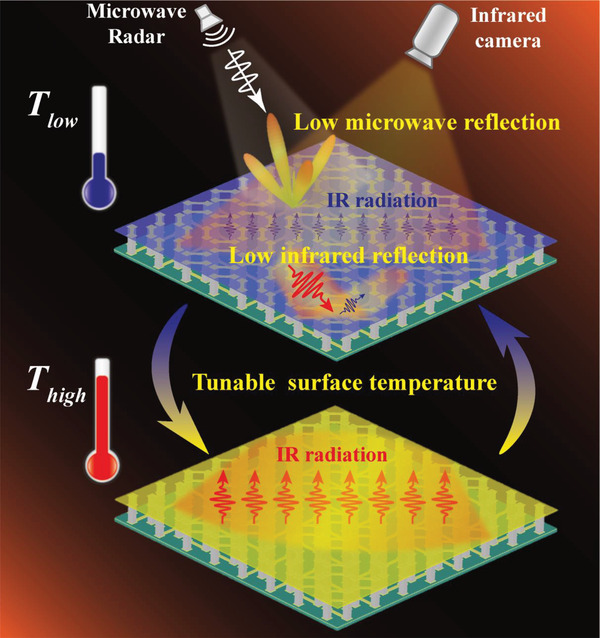
Schematic illustration of the proposed TEMD, which has three functionalities of the microwave scattering reduction, the dynamic control of the surface temperature, and the low IR reflection.

**Figure** **1** schematically illustrates the proposed innovative TEMD, showing its three distinguishing functionalities, i.e., the microwave scattering reduction, the dynamic control of the surface temperature, and the low IR reflection. Our microwave‐IR compatible TEMD utilizes the novel design of the TE pillars along with the electrode structure. Inspired by the principle of the coding metamaterial,^[^ ^30^ ^]^ the basic TEMD element is designed as a geometric phase cell, which can tune the reflection phase by rotating itself with a certain angle. Here, 0° and 180° reflection phase cells are incorporated to construct the whole TEMD by the properly designed arrangement. When microwave is incident onto this TEMD, the backward scattering wave would be forced to propagate in well‐defined ways instead of the specular direction. For instance, the metadevice consisting of a chessboard‐like configuration of elements can redirect the incident wave energy into four diagonal directions corresponding to the azimuth angles of 45°, 135°, 225°, and 315°, cancelling out the specular reflection. Meanwhile, the novel design of the TEMD element has almost no influence on the surface temperature control. When applying the bias current to each TE pillar, the proposed TEMD can realize active cooling and heating for thermal cloaking in the IR range, which can be adaptive to the background temperature change. By further locally controlling the bias current values to the different TE pillars, the pixel surface temperature pattern can be obtained for matching the spatially varying thermal background. Besides, compared with the mostly reported IR camouflage materials using the low‐e coating, the proposed TEMD adopts the high IR‐absorptivity surface instead, which can effectively minimize the reflectance of the surrounding thermal signal and then keep the target away from being detected by the IR camera.

**Figure 2:**
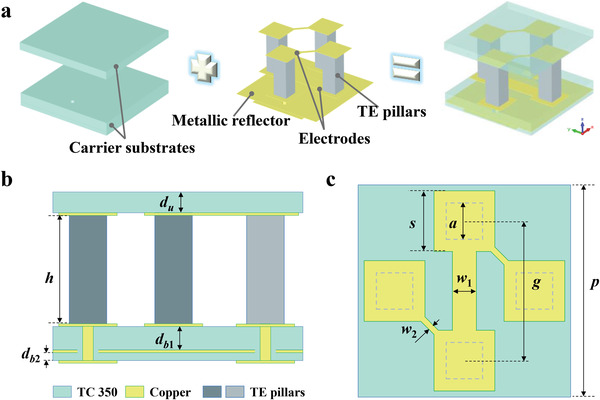
Schematic illustration of the proposed TEMD element. a) Schematic diagram of the basic TEMD element structure. b) Side view of the designed TEMD element in *xoz* plane. c) Top view of the designed TEMD element.

In order to realize the microwave‐IR compatible manipulation, the TEMD element design is crucial, which is schematically illustrated in **Figure** **2**. It is composed of the top and bottom carrier substrates, the sandwiched rigid TE pillars, the electrode structure, and a metallic reflector. The upper electrode with one layer of metallic patterns is carried by the top substrate, while the lower electrode with two layers of metallic patterns accompanied by the metalized via is carried by the bottom substrate, and the metallic reflector is inserted into the bottom substrate, as shown in Figure 2a,b. The TE element has the square shape, and its period is *p*. Four TE pillars with the width of *a* and the height of *h* are distributed symmetrically with respect to the center of the TE element, as shown in Figure 2c. The space between TE pillars is *g* in both the horizontal and vertical directions. The square metallic patterns as a part of the electrode has the width of *s* which is a little larger than *a*, ensuring good electrical connectivity with TE pillars. The widths of the metallic lines connecting the neighbor square metallic patterns in the lower and upper electrodes are *w*_1_ and *w*_2_, respectively. The above element is designed as the “0” element, and the “1” element is obtained by rotating the “0” element by 90° in *x*–*y* plane. The unique binary elements would be used as the two basic geometrical phase cells to realize the 0 and *π* reflection phase responses for microwave coding metadevice design.

**Figure 3:**
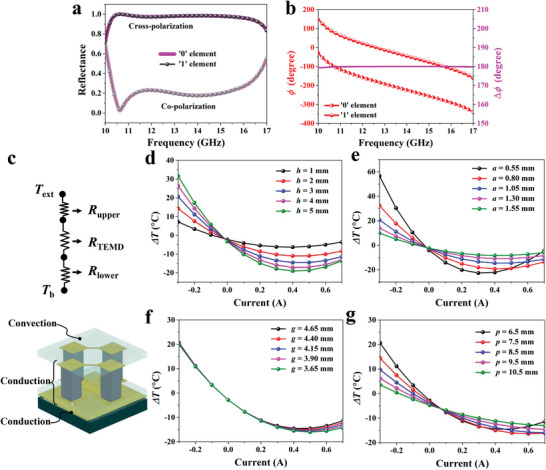
Simulated results of the reflection and thermal performance of the designed TEMD element. a) Simulated co‐ and cross‐polarized reflection magnitude of the binary TEMD elements under the normal linearly polarized illumination. b) Cross‐polarized reflection phase of the binary TEMD elements. c) Thermal resistance model for simulating the TEMD thermal effect. d–g) Simulated results of the temperature difference as a function of the applied current for varying d) the TE pillar height, e) the TE pillar section width, f) the spacing distance, and g) the period.

In this study, we mainly focus on the specular scattering reduction in X—Ku band where many types of radar are strategically deployed for ground target detection. Preliminary simulations indicate that the proper height of TE pillars is about 3.0 mm for good scattering reduction performance in the microwave band, which also gives attention to the thermoelectric effect of TE pillars and mechanical properties. The section width of TE pillars is selected to be 1.05 mm according to commercial productions. They are made of commercial Bi_2_Te_3_ alloys with the electrical conductivity of about 1.2 × 10^5^ S^ ^m^−1^. From the perspective of thermal design, the carrier substrate should have high thermal conductivity, which makes good sense for enhancing the thermoelectric effect and reducing the power consumption.^[^ ^26^, ^42^ ^]^ Here, we adopted Commercial Rogers TC 350 as the top and bottom carrier substrates for the two reasons. One is that its thermal conductivity can reach up to 1.24 W^ ^m^−1 ^K^−1^, and the other is that it is compatible with the printed‐circuit technology for preparing the metallic electrode as well as the metallic reflector. Its dielectric constant and loss tangent are 3.5 and 0.0017, respectively. The other geometrical parameters of the TEMD elements were elaborately optimized as *p* = 6.5 mm, *g* = 3.6 mm, *s* = 1.7 mm, *w*_1_ = 0.2 mm, *w*_2_ = 1.0 mm, *d*_u_ = 0.508 mm; *d_b_*_1_ = 0.508 mm, *d_b_*_2_ = 0.254 mm, respectively. All the thickness of the metallic pattern (deposited copper) of the electrode and the metallic reflector is 17 µm. The diameter of the hole in the metallic reflector is 0.7 mm, which should be larger than the dimension of the metalized via. **Figure** **3** illustrates the simulated results of the reflection and thermal performance of the optimized TEMD element. As shown in Figure 3a, it can be seen that both “0” and “1” elements generate the strong cross‐polarized reflection over the frequency range from 10.2 to 16.7 GHz under the normal linearly polarized illumination, where the reflectance is as high as more than 0.9. Meanwhile, the corresponding phase difference between the cross‐polarized reflection of the above two elements is close to 180° in the whole frequency band of interest, as seen in Figure 3b. Their reflection performances under other incident angles can also be seen in Figure S1, Supporting Information. These results indicate that the designed elements have excellent implementation of the 0 and *π* reflection phase responses. If further assembling the binary TEMD elements into special phase distribution, the microwave scattering reduction could be expected.

For the thermal simulation, we adopted the thermal resistance model,^[^ ^42^ ^]^ in which, heat is exchanged between the target (*T*_b_) and the surrounding (*T*_ext_) through the thermal resistances of the upper side (*R*_upper_), TEMD (*R*_TEMD_), and the lower side (*R*_lower_), as illustrated in Figure 3c. Note that the *R*_lower_ includes the thermal contact resistance at the interface between the TEMD and the target, which is increased significantly due to the existence of microscale unevenness of solid surface (see Figure S2, Supporting Information). The heat transfer at the air side depends on the natural convection, and the conduction is considered both inside and at the bottom side of our TEMD. The surface temperature modulation, which is represented by the difference between the average temperature at the air side and the surface temperature of the target (Δ*T*), is elaborated in details in Figure 3d–g for various structural parameters, including the height and the section width of the TE pillar, the spacing distance as well as the period. In general, the heating effect is enhanced with the increase of the applied current, while there is an optimum value for the applied current to maximize the cooling effect. As shown in Figure 3d,e, it can be found that the long and slender shape of the TE pillar is beneficial to the surface temperature modulation. However, excessively longer and more slender pillars would lead to the larger electrical resistance inducing more Joule heat. In addition, the mechanical strength of the TEMD would be also degraded because of the fragility of the TE pillar. The spacing distance has little influence on the heating performance, but it would slightly affect the cooling performance, as shown in Figure 3f. Besides, we also investigated the influence of the spacing distance on the surface temperature uniformity, as shown in Figure S3, Supporting Information. It can be observed that the spacing distance has slight effect on the surface temperature uniformity. As the period increases, the thermal conductance between the cold and hot sides of the TEMD decreases, resulting in that the best cooling effect is enhanced a little but the power consumption increases, as illustrated in Figure 3g. Based on the above simulation results, it can be concluded that the designed TEMD element can simultaneously meet the dual requirements of the reflection phase control and the surface temperature modulation, thus paving the way to achieve microwave‐IR compatible manipulation.

**Figure 4:**
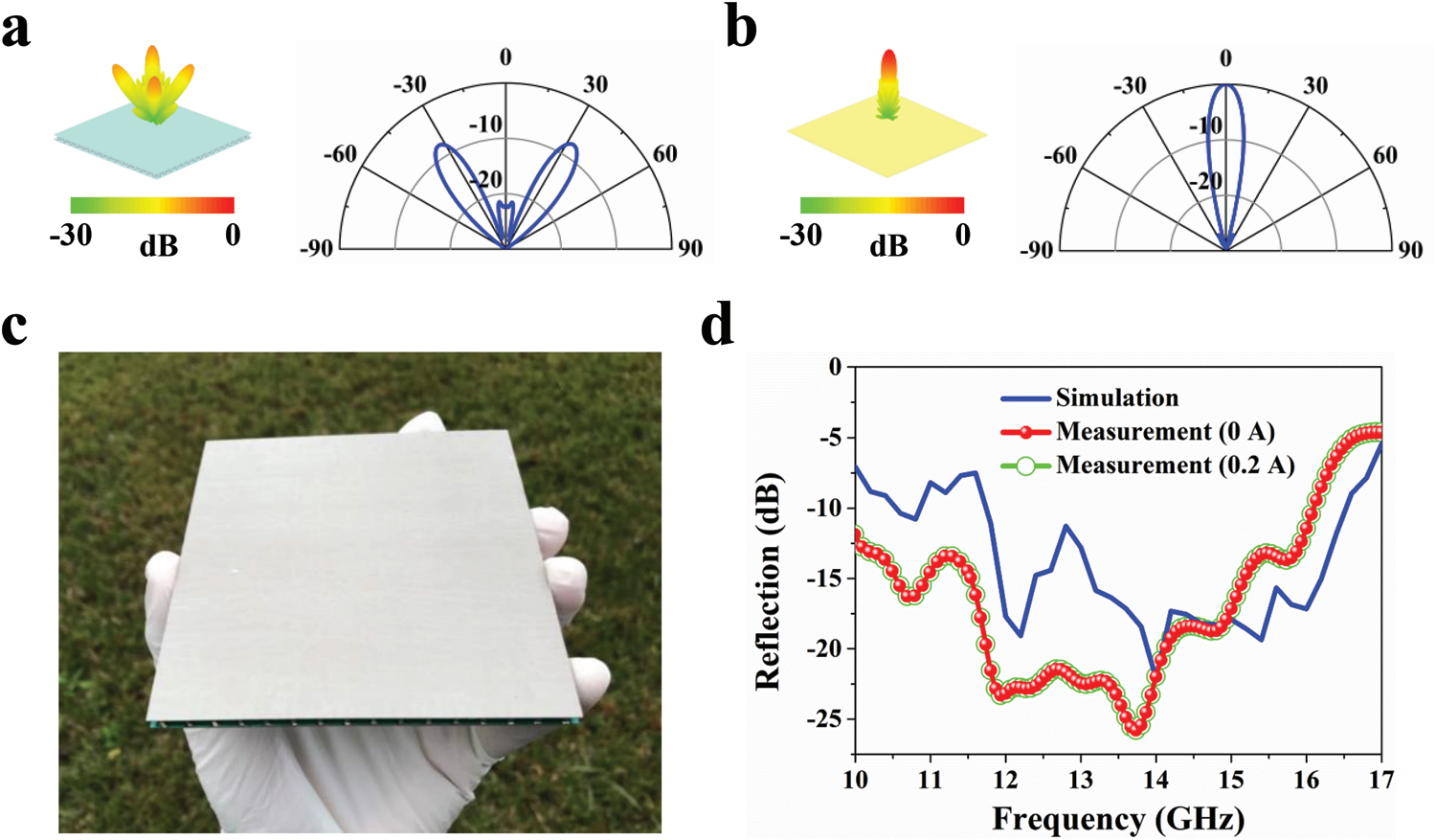
Microwave reflection performance of our TEMD. a,b) Simulated results of the normalized 3D scattering patterns accompanied by normalized 2D scattering patterns in the 45° plane for a) our TEMD and b) a same‐size metallic plate at 14 GHz, respectively. c) Photograph of the fabricated TEMD. d) Measured and simulated reflection level of our TEMD under the normal illumination. Measured results were obtained under supplying bias currents of 0 and 0.2 A to our TEMD, respectively.

Subsequently, the TEMD is constructed by using the binary elements with the special phase arrangement. Here, the demonstrated model is composed of 4 × 4 subgroups distributed in the chessboard‐like configuration. Each subgroup is made of 4 × 4 basic elements, i.e., the “0” or “1” element, to ensure the electromagnetic similarity between the subgroup and the corresponding basic element. Full‐wave simulation was performed to quantitatively evaluate the microwave scattering reduction performance. For comparison, a metallic plate with the same dimension was also simulated. **Figure** **4**a,b illustrates the scattering patterns of our TEMD and the metallic plate under normal incidence at a representative frequency (14 GHz), respectively. It can be observed that our TEMD makes the microwave scattering energy redirected into four symmetrically oriented directions in the 45° and 135° planes, while almost all the incident energy is reflected into the normal direction by the metallic plate. By comparison, our TEMD sharply suppresses the reflection, and its reflection level is only about −22 dB. More simulation results of the scattering pattern at other frequencies are illustrated in Figure S4, Supporting Information. In actual applications, it is an effective strategy for microwave stealth to force the scattering waves to deviate from the direction of the surveillance radar. Furthermore, it is worth noting that our TEMD can also realize the diffusion scattering by further optimization of the coding sequence of the binary TE elements, which can suppress the scattering energies to the low levels in all directions (see Figure S5, Supporting Information).

To experimentally demonstrate the performance of the microwave scattering reduction, we fabricated the TEMD according to the above simulation model, as shown in Figure 4c. The microwave reflection performance was measured in a microwave anechoic chamber (see Figure S6, Supporting Information). Figure 4d shows the measured reflection performance, which also includes the simulated results for comparison. It is observed that our TEMD device can realize −10 dB reflection level over a wide frequency range of 10–16.1 GHz, and especially in 11.7–14.1 GHz, the reflection level is sharply reduced to about 20 dB. In addition, it is found that applying the bias current to our TEMD has no influence on its microwave reflection property. The measured reflection curve agrees well with the simulated one, although there is a slight frequency shift due to the fabrication tolerance. In addition, the reflection performances of the sample under oblique incidence were also discussed (see Figure S7, Supporting Information). All the measured results reveal that our TEMD has a good angular stability, which can effectively suppress the specular reflection wave in the broadband frequency range. Besides, we also measured the scattering pattern of our TEMD (see Figure S8, Supporting Information). The measured results agree with the simulated ones in general. Note that the scattering reduction bandwidth is limited for our TEMD. If further broadening the bandwidth, more basic elements are required to generate various reflection phases, which could increase the ability to achieve phase cancellation. Here, more basic elements could be constructed by using the TE elements with different rotating angles or adopting multilayer TE structure design, and then the planar array theory combined with optimization algorithm could be employed to obtain the optimal arrangement of all the basic elements,^[^ ^43^ ^]^ which may realize super‐wideband scattering reduction.

**Figure 5:**
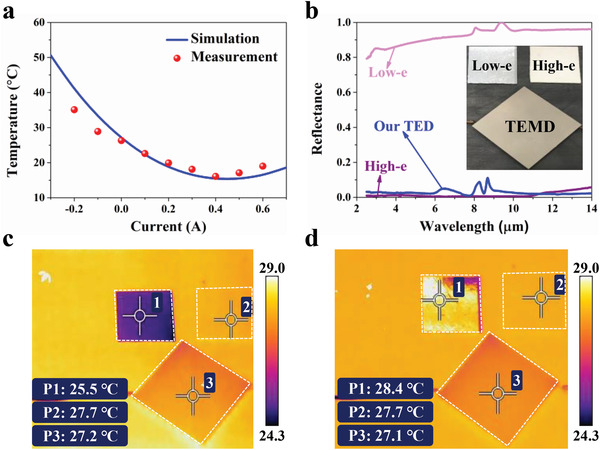
a) Measured and simulated results of the average temperature at the air side of our TEMD as a function of the applied current. b) Measured spectral reflectance of our TEMD along with a low‐e Al foil and a high‐e ceramic wafer for comparison. The inset shows photographs of our TEMD, the Al foil, and the ceramic wafer. c) The IR snapshots of the three samples when all the lamps in the room were switched off. d) The IR snapshots of the three samples when all the lamps were switched on. The bright (yellow) spot on the Al foil surface shows the reflection of the thermal signal from the nearby lamps.

After the experimental demonstration of the microwave functionality of our TEMD, we subsequently investigated its thermal performance for the dynamic IR camouflage. The TEMD sample was placed on a thermostat stage, and its surface temperature can be measured by a thermocouple under different applied currents (see the measurement setup in Figure S9a,b, Supporting Information). During the measurement, the temperature of the thermostat stage was set to be 30 °C and the room temperature was 24 °C. As **Figure** **5**a shows, the measured minimum and maximum surface temperatures are 16.1 and 35.1 °C when supplying our TEMD with the bias current of 0.4 and −0.2 A, respectively. The temperature tuning range reaches up to 19 °C. According to the experimental results of the bias voltage (*V*) and the applied current (*I*), the fitting electrical resistance (*R*) of our TEMD is about 42.2 Ω, and then the consumption power can be calculated by *P = I* ^2^ *R*, which is about 6.75 W at the bias current of 0.4 A, as shown in Figure S9c,d, Supporting Information. It is worth pointing out that the maximum surface temperature of our TEMD can be further elevated by increasing the reversed bias current, which can further broaden the surface temperature tuning range. In addition, the different target temperature and the ambient temperature have great influence on the surface temperature value, but the temperature tuning range remains almost unchanged (see Figure S10, Supporting Information). In our TEMD, the high‐emissivity (high‐e) surface is utilized instead of the low‐e material. Although the low‐e material can effectively suppress the thermal radiation from the target, it also means high IR reflectance, which would lead to the target being captured by an IR detector through the reflection of the surrounding thermal signals. To verify this phenomenon, we adopted a low‐e Al foil and a high‐e ceramic wafer to compare with our TEMD sample. Over the wavelength range from 3 to 14 µm, the low‐e Al foil has an average reflectance of 0.930, while it is 0.015 (close to 0.028 for our TEMD sample) for the high‐e ceramic wafer, as shown in Figure 5b. When all the lamps in the room were switched off, the low‐e Al foil has obviously lower apparent temperature, as shown in Figure 5c. However, when all the surrounding lamps were turned on, the apparent temperature of the low‐e Al foil comes to be higher due to the reflection of the thermal radiation from these lamps, as seen in Figure 5d. This result implies the imperfection of low‐e material. Both the ceramic wafer and our TEMD can suppress the reflection of the thermal signals from the hot lamps. Meanwhile, our TEMD also displays a little lower apparent temperature due to the thermal resistance of its sandwiched structure.

**Figure 6:**
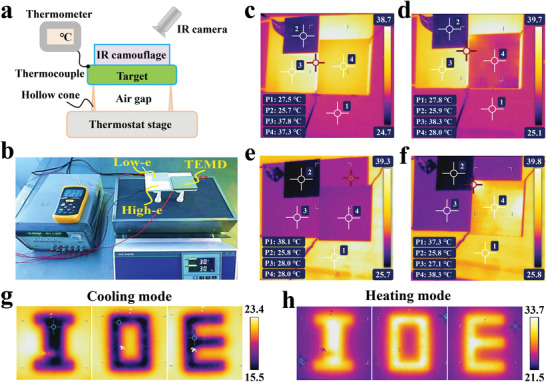
Experimental demonstration of dynamic IR‐camouflage effect of our TEMD. a) Schematic diagram to verify the IR‐camouflage performance by an IR camera. b) Photograph of the measurement setup. c,d) At the low‐temperature background, the IR images of the Al foil, the ceramic wafer, and our TEMD sample c) without and d) with the bias current of 0.14 A. e,f) At the high‐temperature background, the IR images of the Al foil, the ceramic wafer, and our TEMD sample e) without and f) with the bias current of −0.11 A. g,h) IR images of the letters “I,” “O,” and “E” by pixel manipulation with the different bias current configurations at the g) cooling and h) heating modes.

In order to verify the dynamic IR camouflage performance of our TEMD, we first demonstrated its capability of modulating the surface temperature to match the background temperature. In this experiment, we employed a commercial TED and the thermostat stage as the target and the background, respectively. The target was separated from the background by four hollow plastic cones, ensuring the essential thermal isolation between the target and the background, as illustrated in **Figure** **6**a. The experimental setup is shown in Figure 6b. The heating plate of the thermostat stage is composed of graphite whose IR emissivity is close to 1. Besides, employing the thermostat stage as the background is owing to its excellent uniformity and easy manipulation of the surface temperature. The target was covered by our TEMD as well as the low‐e Al foil and the high‐e ceramic wafer for comparison. An IR camera was utilized to record the IR images. Two different scenarios were imitated in the experiment. One is that the surface temperature of the target was heated up to 40 °C, which is higher than the background temperature (30 °C), while the other is the reverse, i.e., the target surface temperature (30 °C) is lower than the background temperature (40 °C). The recorded IR images are illustrated in Figure 6c–f, respectively. As shown in Figure 6c, the apparent temperature covered by the low‐e Al foil is close to the background temperature, indicating its camouflage effect in this scenario. The high‐e ceramic wafer has no camouflage effect due to its high thermal conductivity and high emissivity. Although our TEMD also has the high‐e surface, it can modulate its apparent temperature to match the background temperature by applying the bias current of 0.14 A, as shown in Figure 6d. In the other scenario, the ceramic wafer still has no camouflage effect, while the low‐e Al foil brings the negative impact on the IR camouflage, and more significant difference of the apparent temperature between the target and the background is observed in Figure 6e,f. Because the background temperature is obviously larger than the target temperature, and the surface emissivity of the background is very high (≈1), it is impossible to enhance the IR radiation intensity of the target to match the background by further increasing the surface emissivity, which cannot exceed 1 for all the materials. That means the emissivity manipulation approach is no longer valid for the IR camouflage in this scenario. Nevertheless, it is seen in Figure 6f that our TEMD possesses the capability of dynamically modulating the IR appearance, and its apparent temperature can be tuned to match the high‐temperature background by applying the bias current of −0.11 A. Meanwhile, the transient temperature change was recorded in Figure S11 of the Supporting Information, and about 100 s is required for our TEMD to reach the target temperature at both the heating and cooling modes. In order to further increase the response speed, the strategy of applying the step‐wise current, i.e., high current at first and then low current, can be adopted to quickly approach the desired temperature.^[^ ^28^ ^]^ In the end, we also verified the pixel‐manipulation capability of our TEMD on the surface temperature distribution. As Figure 6g,h shows, the TEMD sample displays the IR image of letters “I,” “O,” and “E” (the initials of Institute of Optics and Electronics) at the heating and cooling modes, respectively. The lower electrode can be patterned to area‐selectively control the current of corresponding TE pillars. When supply 0.2 A to the pixels of letters “I,” “O,” and “E,” the low‐temperature pixels are obtained, as remarkably displayed in Figure 6g. After reverse the applied current, i.e., −0.2 A, the temperature of the pixels comes to be higher, as shown in Figure 6h. If supplying the different currents to the pixels of letters “I,” “O,” and “E,” we can further obtain the IR images with gradient colors for displaying each letter, as seen in Figure S12, Supporting Information. These results indicate that the surface temperature of our TEMD can be modulated by pixel, which means that our TEMD can be utilized for adapting to a spatially varying thermal background. The above results have fully demonstrated that the proposed TEMD has the good IR camouflage property in various backgrounds.

## 3 Conclusion

In summary, an innovative TEMD is proposed which can not only reduce the microwave specular scattering based on the geometrical phase modulation, but also achieve dynamic IR camouflage by tuning the surface temperature along with minimizing the reflection of surrounding thermal signals. A proof‐of‐concept device is fabricated to experimentally demonstrate its microwave and IR performance. Measurement results reveal that our TEMD can not only realize the 10 dB reflection reduction over the frequency range from 10 to 16.1 GHz, but also dynamically modulate the surface temperature in a broad tuning range for matching with various high‐ and low‐temperature backgrounds. Meanwhile, it is also observed that our TEMD reflects almost no surrounding thermal signals compared with the traditional low‐e material. Besides, the surface temperature of our TEMD can be further modulated by pixel for adapting to the spatially varying thermal background. The fabrication of our TEMD mainly depends on the existing printed‐circuit technology and TED packaging technology, and thus it is easy to achieve mass production with low cost. It is the first time to introduce the concept of coding metamaterial into the TED design, which makes this traditional device revitalized. We believe that such design provides an effective approach to achieve microwave‐IR compatible manipulation, which may find much potential application in multispectral camouflage and stealth fields.

## 4 Experimental Section

### Fabrications

The metallic patterns (17 µm thickness copper) on the carrier substrate were etched by the printed‐circuit technology. To prevent oxidation, gold coating with the thickness of several nanometers was deposited on the copper patterns. In order to ensure the TE pillars aligning with the electrode, a pre‐prepared mold was utilized, which was composed of arrays of rectangle blind holes corresponding to the pillar distribution. The TE pillars and the electrode were soldered together by heating the carrier substrate to finish the fabrication of TEMD. The fabrication process can be found in Figure S13, Supporting Information.

### Characterizations

The microwave reflection characteristic of the fabricated TEMD was measured under linearly polarized illumination in a calibrated NRL arch using an Agilent N5224A vector network analyzer. The incidence and reflection angle could be modulated by the electric motor. To approximate the normal incidence condition, both the incidence and reflection angles were fixed as 5°. The distance from the TEMD to the transmitting or receiving antenna was about 3.5 m, which was far enough to avoid the near field effect. The transmitting horn generated the quasi‐plane wave to illuminate the sample and the reflected wave energy was captured by the receiving horn. The microwave scattering pattern measurement was performed in a microwave anechoic chamber. The transmitting horn was placed at a distance of 1.5 m in front of the TEMD sample. Both the sample and the transmitting horn were fixed on a mechanical rotary table that was controlled by software in computer. When rotating the rotary table, the sample was always illuminated by the normal incident wave produced by the transmitting horn, and the reflected wave signal for the (−90° to +90°) rotating angles could be received by the receiving horn antenna with the angular resolution of 0.1°. The IR reflectance of the carrier substrate, the Al foil, and the ceramic wafer was measured using the IR Fourier Spectrometer VERTEX80. IR images were recorded by FLIR T650sc with a spectral range of 7.5–13.0 µm.

### Statistical Analysis

The fitting electrical resistance was obtained using the least square method. The simulated RCS of the metadevice was baseline subtracted and normalized by that of the same‐sized PEC plate. Other data were presented as raw data.

## Conflict of Interest

The authors declare no conflict of interest.

## Supporting information

Supporting Information

## Data Availability

The data that support the findings of this study are available from the corresponding author upon reasonable request.
