# Supplementary material for: A Dynamic Thermal Camouflage Metadevice with Microwave Scattering Reduction
Source: Adv Sci (Weinh). 2022 Jun 5;9(22):2201054. doi: 10.1002/advs.202201054 (PMC9353466; doi:10.1002/advs.202201054)
Supplement: Supplementary file 1 — Supporting Information [file ADVS-9-2201054-s001.pdf]

ADVANCED SCIENCE Open Access

Supporting Information

for *Adv. Sci.*, DOI 10.1002/advs.202201054

A Dynamic Thermal Camouflage Metadevice with Microwave Scattering Reduction

*Liming Yuan, Cheng Huang, Jianming Liao, Chen Ji, Jingkai Huang, Yuetang Wang and Xiangang Luo**

Supporting Information

A Dynamic Thermal Camouflage Metadevice with Microwave Scattering Reduction

*Liming Yuan, Cheng Huang, Jianming Liao, Chen Ji, Jingkai Huang, Yuetang Wang, and Xiangang Luo**

S1. Simulated results of the microwave reflection performance for the TEMD elements under different incident angles.

Figure S1a illustrates the simulated cross-polarized reflection characteristics of the '0' and '1' TEMD elements under linearly-polarized illuminations with the different incident angles. It can be seen that the binary elements have the cross-polarization reflectance of more than 0.9 over the frequency range from 10.2 to 16.0 GHz for all the incident angles. Meanwhile, the relative difference between the cross-polarized reflection phases of the binary elements under different incident angles is close to 180° in most of the above frequency bands, as shown in Figure S1b.

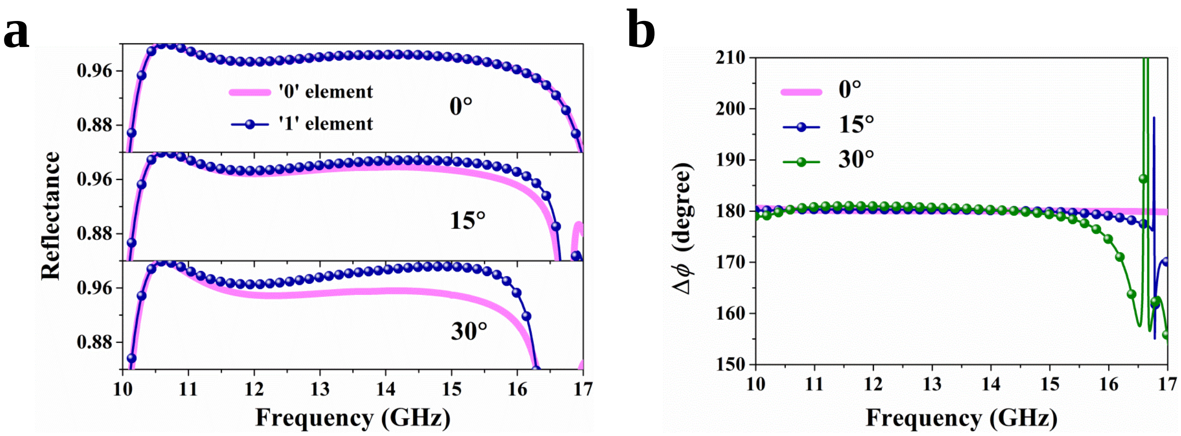

Figure S1. Simulated results of the reflection performance of the designed TEMD element. (a) Simulated cross-polarized reflection magnitude of the binary TEMD elements under the normal linearly-polarized illumination for different incident angles. (b) Relative difference between the cross-polarized reflection phase of the '0' and '1' elements for different incident angles.

S2. Thermal simulation for the TEMD element.

Heat is exchanged between the target and the surrounding through the thermal resistances of the upper side, TEMD and the lower side. The thermal resistance of the lower side includes the thermal contact resistance (R_{TC}) at the interface between the TEMD and the target. Because of the existence of micro-scale unevenness of solid surface, the real contact area is smaller than that of the nominal contact surface area, as shown in Figure S2, resulting in a significant increase in R_{TC}, which could be expressed as follow

$$R_{TC} = \frac{\sigma/m}{1.25A\kappa_s\left(P/H_c\right)^{0.95}} \tag{1}$$

in which, σ, m, A, κ_S, P and H_C are the root mean square of surface roughness, the average surface asperity slope, the macroscopic contact area, the harmonic mean thermal conductivity, the applied pressure and the microhardness of the substrate, respectively. The equivalent roughness, surface slope and thermal conductivity can be calculated by

$$\sigma = \sqrt{\sigma_1^2 + \sigma_2^2} \tag{2}$$

$$m = \sqrt{m_1^2 + m_2^2} \tag{3}$$

$$\kappa_s = \frac{2\kappa_1\kappa_2}{\kappa_1 + \kappa_2} \tag{4}$$

where the number '1' and '2' represent the bottom substrate of our TEMD and the target, respectively. In our thermal simulations, the values of σ, m, P, H_C and the convection coefficient at the air side are 1 μm, 0.4, 100 kPa, 3 Gpa and 25 W m^{-2} K^{-1}, respectively.

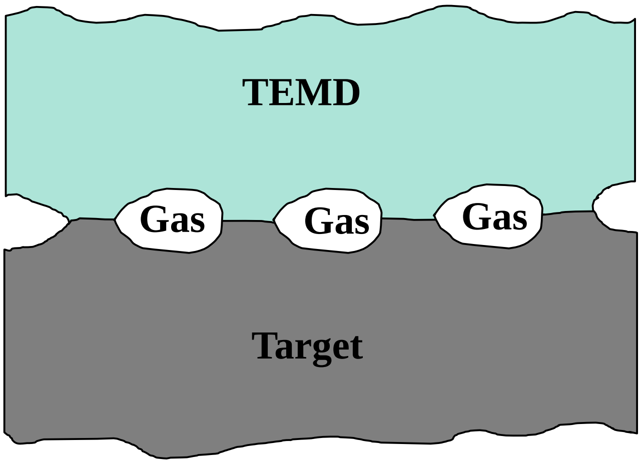

Figure S2. The model of the interface between our TEMD and the target.

The spacing distance may lead to non-uniform temperature distribution. For this purpose, we investigated the surface temperature distribution of our TEMD element under the identical average temperature for different spacing distances. In the simulation, the average surface temperature of the TEMD element was set to be 10°C higher and lower than the temperature of the target (30°C) at the heating and cooling modes, respectively. The Simulated air-side surface temperature distribution is illustrated in Figure S3. It can be observed that the higher the surface temperature is, the larger the maximum variation is, i.e., the maximum temperature variation at the heating mode is larger than that at the cooling mode for the same TEMD element. At the heating mode, the maximum temperature variation changes from 1.26°C to 1.16°C, while it slightly changed from 0.32°C to 0.29°C at the cooling mode, as the spacing distance increased from 3.65 mm to 4.65 mm. The above result shows that the

spacing distance in our design has slight influence on the surface temperature uniformity, which may provide more freedom to optimize the microwave scattering reduction performance.

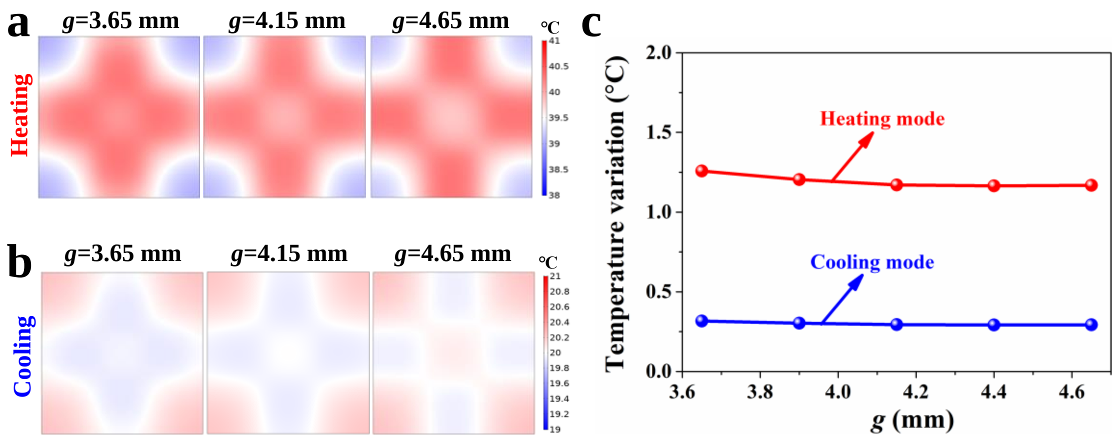

Figure S3. Influence of the spacing distance on the temperature distribution at the air-side of the TEMD element. (a) Simulated results at the heating mode. (b) Simulated results at the cooling mode. (c) The maximum temperature variation for the different spacing distances.

S3. Microwave scattering simulation of the designed TEMD

The full-wave simulation model of the designed TEMD is composed of 4×4 subgroups distributed in the chessboard-like configuration. Each subgroup is made of 4×4 basic elements, i.e., the '0' or '1' element, to ensure the electromagnetic similarity between the subgroup and the corresponding basic element. The TEMD is illuminated by the normal linearly-polarized wave at the frequency band of 10~17 GHz. Simulated results of the scattering patterns of our TEMD are displayed in Figure S4. Here, the scattering magnitude is normalized by the PEC plate with the same size. As expected, the incident microwave is mainly redirected into four symmetrically oriented directions at the effective response frequency, resulting in greatly suppressing the specular scattering. The direction (deflection angle θ, azimuth angle β) of the anomalous scattering waves can be calculated by the following equations:

$$\theta = \arcsin\left(\lambda\sqrt{\frac{1}{L_x} + \frac{1}{L_y}}\right) \quad (1)$$

$$\beta = \pm\arctan\frac{L_x}{L_y}, \pi \pm \arctan\frac{L_x}{L_y} \quad (2)$$

in which, λ is the wavelength of the incident wave; L_x and L_y are the periodic lengths along x- and y-direction, respectively. For the constructed model, $L_x = L_y = 8p = 52$ mm. It can be seen that the scattering elevation angle at 15 GHz is about 30°, which is consistent with the theoretical calculation (33°) by Equation (1). As shown in the figure, it could be seen that the more high-efficiency polarization conversion the binary elements has, the greater reflection reduction our TEMD can obtain.

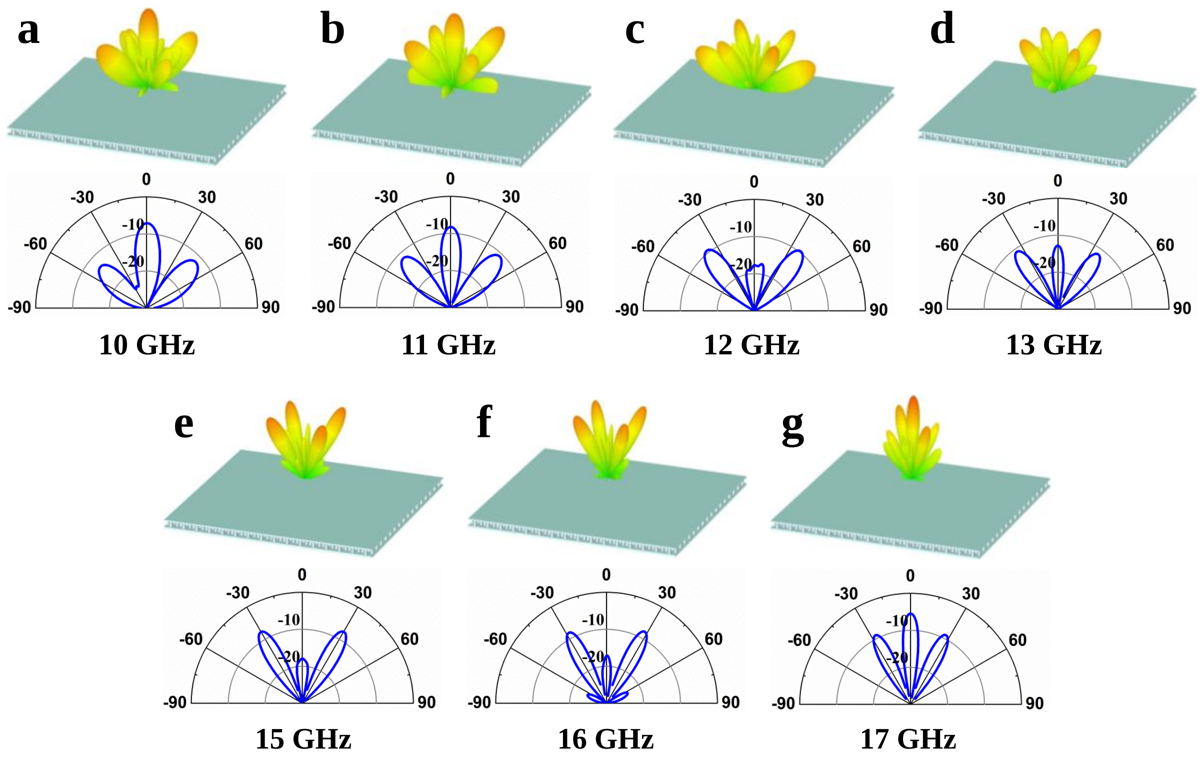

Figure S4. Simulation results of the normalized 3D scattering pattern accompanied by the normalized 2D scattering pattern in the 45° plane for our TEMD at (a) 10 GHz, (b) 11 GHz, (c) 12 GHz, (d) 13 GHz, (e) 15 GHz, (f) 16 GHz and (g) 17 GHz, respectively.

In order to achieve the better microwave scattering performance, the arrangement of the binary TEMD elements can be further optimized through genetic algorithm, as shown in

Figure S5. The simulated specular reflection of the TEMD is given in Figure S5b, which also includes the same-size PEC plate for comparison. It is noted that the TEMD with the optimized phase distribution can achieve the 10-dB scattering reduction over a wide frequency band of 8.5~16.5 GHz, except for the frequency of around 11 GHz where about 9-dB scattering reduction is obtained. Figure S5c~f show the 3D scattering patterns of the TEMD at the four representative frequencies of 10 GHz, 12 GHz, 14 GHz and 16 GHz, respectively. It is found that the TEMD can diffuse the backscattering wave to all directions, and the scattering energy in each direction is very small, achieving significant reductions in both monostatic and bistatic RCSs.

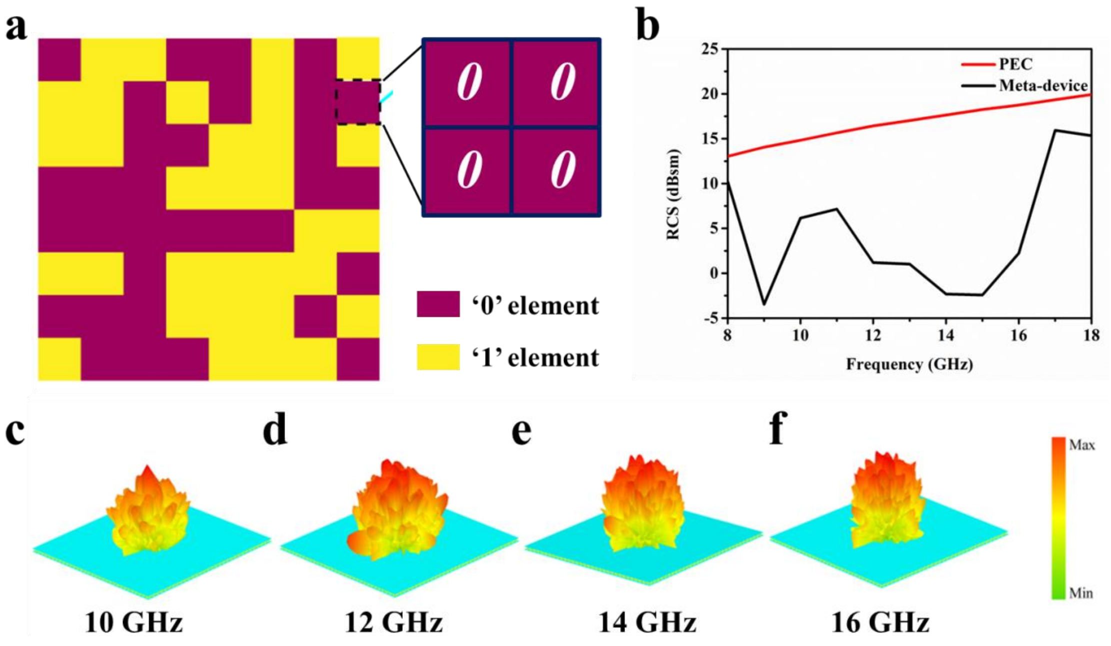

Figure S5. (a) Schematic illustration of the TEMD with the optimized coding sequence for realizing diffusion scattering. (b) Simulated specular reflection reduction performance of the optimized TEMD. (c~f) Simulated scattering patterns of the optimized TEMD at (c) 10 GHz, (d) 12 GHz, (e) 14 GHz and (f) 16 GHz, respectively.

S4. Experimental demonstration for the microwave reflection reduction

The microwave reflection characteristic of the TEMD sample is measured by using the arch

measurement system, as seen in Figure S6. Two linearly polarized standard horn antennas respectively set as receiver and transmitter are placed on an arch range and the sample is located at its circle center. Figure S7 shows the measured results of the TEMD sample under the TE and TM polarized illuminations with different oblique incident angles. It can be observed that our TEMD sample can still achieve low reflection property in the frequency band of 10~16.1 GHz when the incident angle varies from 10° to 30° under both TE and TM polarizations. Although the increase of the incident angle makes the -10 dB reflection bandwidth decrease a little, most of the reflection levels are still about -7~-10 dB. The results indicate that our TEMD sample may have a good angular stability.

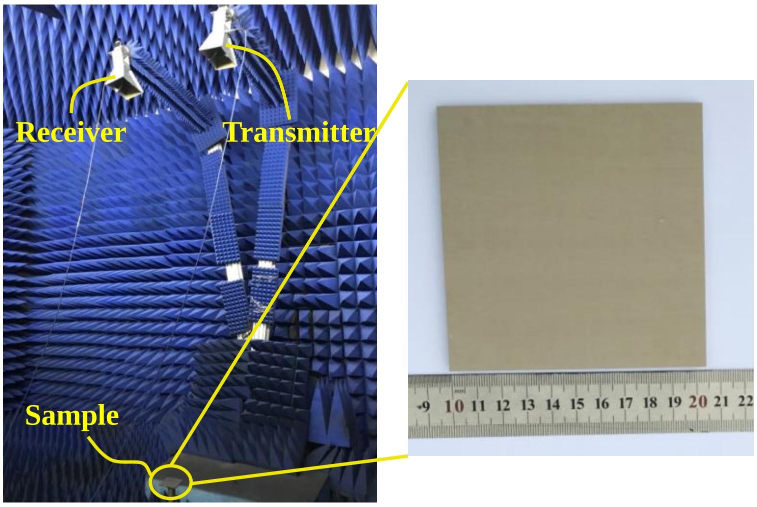

Figure S6. Photograph of the experimental setup for measuring the microwave reflection performance of the fabricated TEMD sample.

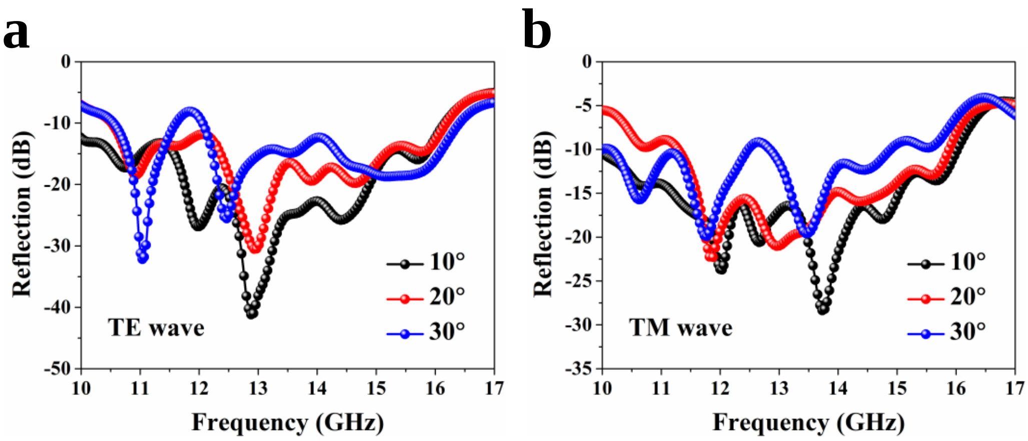

Figure S7. Measured microwave reflection performance under the different oblique incident angles. (a) TE polarized illumination. (b) TM polarized illumination.

We also measured the backward scattering pattern of our TEMD sample in the 45° plane, as shown in Figure S8. It can be seen that the backscattering wave is redirected into the pre-designed directions and then the specular reflection is sharply suppress. The measured results agree with the simulated ones in general. Some deviation between them is mainly caused by the fabrication tolerance and the measurement error. In addition, the measurement environment also has some difference from the simulation condition, for instance, the ideal plane wave in the simulation is difficult to be realized by the horn antenna in the experiment.

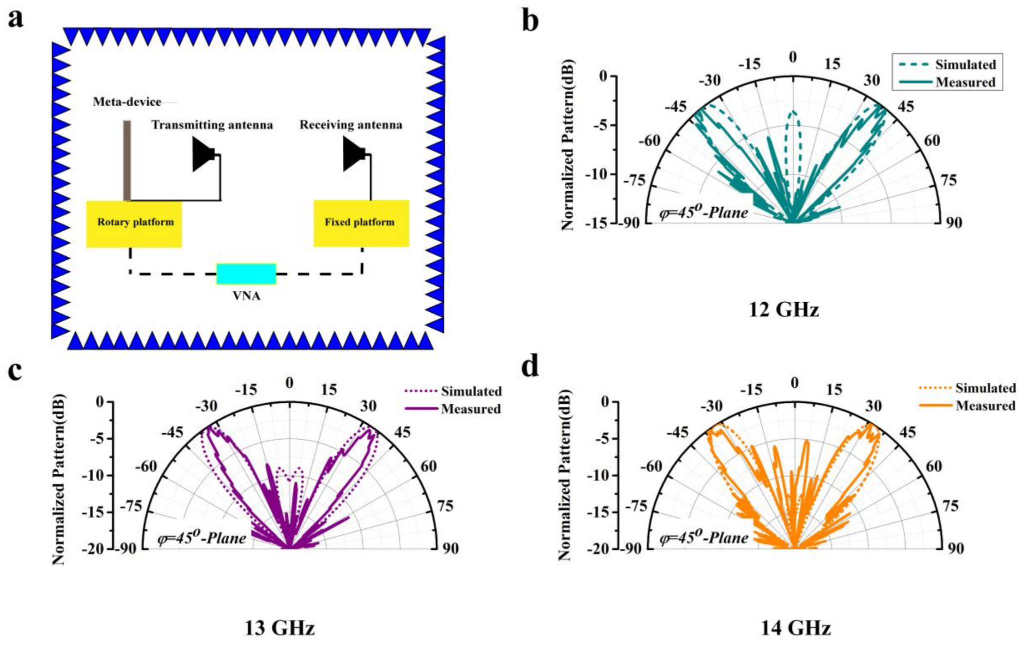

Figure S8. (a) Schematic illustration of the experimental setup for the backward scattering pattern measurement. (b~d) Measured and simulated results of the normalized 2D scattering pattern in the 45° plane at the representative frequencies of (b) 12 GHz, (c) 13 GHz and (d) 14 GHz, respectively.

S5. Study on the surface temperature modulation

The cooling and heating effects of our TEMD are characterized by aid of a thermocouple and a thermostat stage, as shown in Figure S9a,b. The thermostat stage is fixed at a constant

temperature, and the TEMD surface temperatures under the different applied currents are recorded by the thermometer. A DC source is utilized to supply our TEMD sample current. The bias voltages (***V***) along with the corresponding currents (***I***) were also recorded for extracting the electrical resistance (***R***), as shown in Figure S9c. Then the consumption power (***P***) can be calculated by $P=I^2R$ under various applied currents, as shown in Figure S9d.

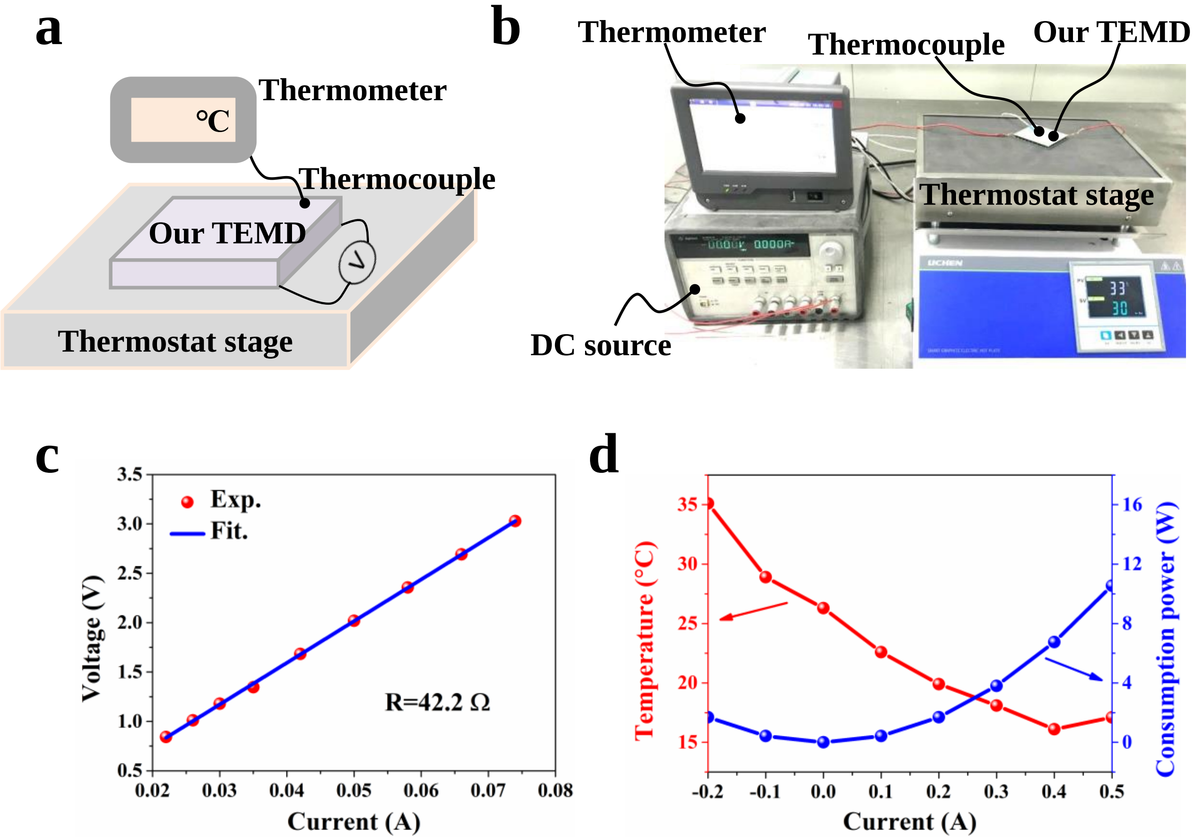

Figure S9. (a) Schematic diagram to measure the air-side surface temperature of our TEMD. (b) Photograph of the measurement setup. (c) Experimental results of the bias voltage as a function of the applied current and the fitting result of the electrical resistance of our TEMD. (d) Measured results of the temperature at the air side along with the calculated results of the consumed power as a function of the applied current.

The surface temperature of the TEMD is related to the temperature of the target as well as the surrounding air. Here, we simulated our TEMD under various temperatures of the target and the surrounding air, respectively, as shown in Figure S10. It can be seen that all the simulated curves have the very consistent trend, meaning that the surface temperature tuning

range almost remains unchanged. For all the simulated cases, the maximum cooling effect occurs at the applied current of about 0.4 A. When the applied current changes from -0.4 A to 0.4 A, the surface temperature tuning ranges of our TEMD for all cases are close to 50 °C.

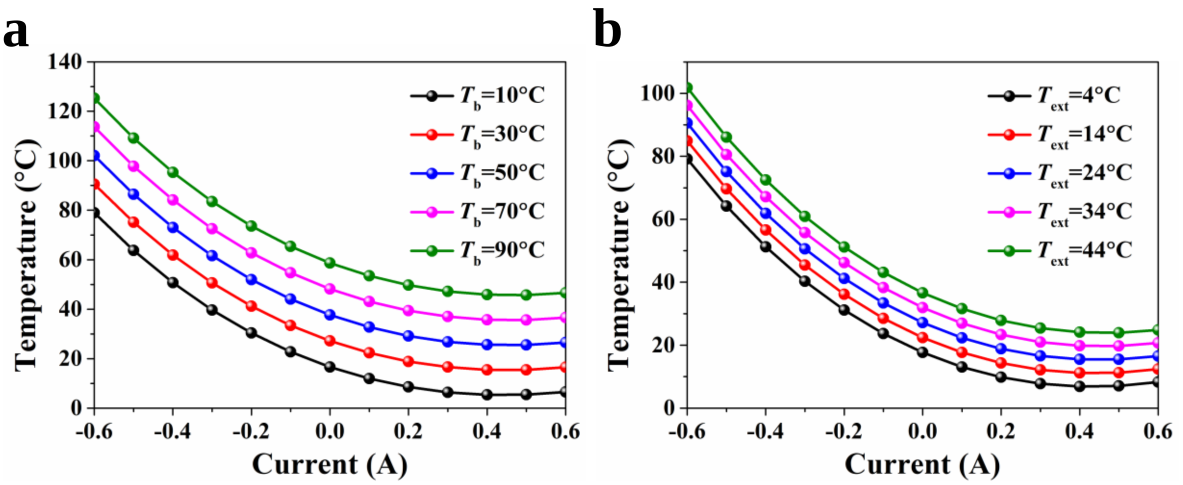

Figure S10. Simulated surface temperatures as the function of the applied current for various temperatures of (a) the target and (b) the ambient air.

The transient temperature change was recorded in the measurement for the heating mode under the external current of -0.11 A and the cooling mode under the external current of 0.14 A, respectively. As Figure S11 shows, it takes about 100s for our TEMD to reach the target temperature in both heating and cooling modes.

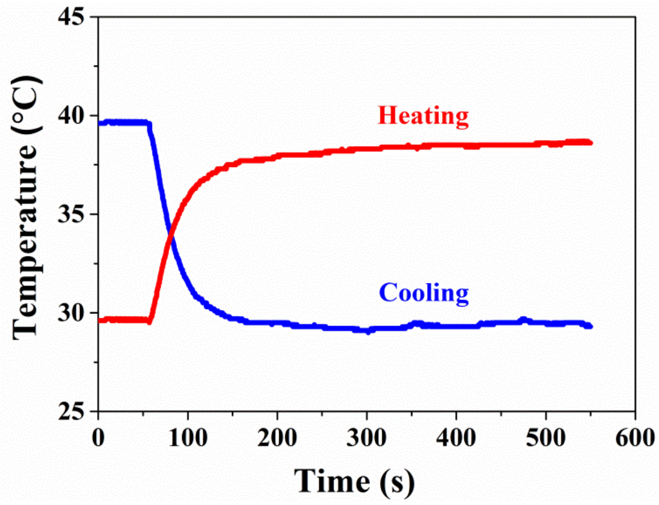

Figure S11. Measured transient temperature change of our TEMD for the heating and cooling modes.

S6. The demonstration of the pixel temperature control

Here, we supplied the different bias currents to the pixels of the letter 'I', 'O' and 'E'. Details on the loaded currents are given in Figure S12. It can be seen that the IR image of each letter display the gradient colors. This result further demonstrates that our TEMD has the pixel-manipulation capability of controlling the surface temperature.

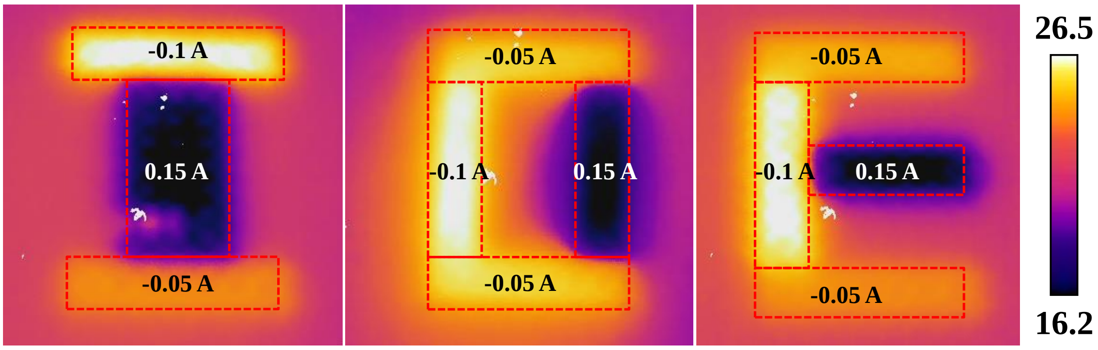

Figure S12. IR images with gradient colors of the letters 'I', 'O' and 'E' by supplying different currents to the pixels.

S7. Fabrication process of our TEMD

As illustrated in Figure S13, the process of fabricating our TEMD mainly includes the following six steps: Firstly, the metallic patterns on the top and bottom carrier substrates are etched by the printed-circuit technology. The top carrier substrate includes one layer of metallic patterns and the bottom carrier substrate includes three layers of metallic patterns, one of which is the microwave reflector. To prevent oxidation, the gold coating with the thickness of several nanometers is deposited on the copper patterns. Secondly, the electrode on the bottom carrier substrate is covered with solder paste by the aid of a pre-prepared metal mesh, which is fabricated according to the electrode. Thirdly, the TE pillars (1.05 mm × 1.05 mm in cross section and 3 mm in height) are put into a pre-prepared mold, which is composed of arrays of rectangle blind holes corresponding to the pillar distribution of our TEMD. After all the TE pillars are filled in the holes, they are covered by the bottom carrier substrate,

ensuring that they are aligned with the electrode. Fourthly, the bottom carrier substrate is heated to solder the TE pillars with the electrode under the conduction heat. Since Rogers TC 350 with good thermal conductivity is used as the carrier substrate, heat can be conducted efficiently by the carrier substrate. Fifthly, after all the TE pillars are soldered completely with the electrode, they are pull out from the mold, and then they are covered with the top carrier substrate, on which, the electrode has been coated by the solder paste through the same process described in the second step. Finally, the top carrier substrate is heated to solder the TE pillars with the electrode to finish the fabrication of our TEMD sample.

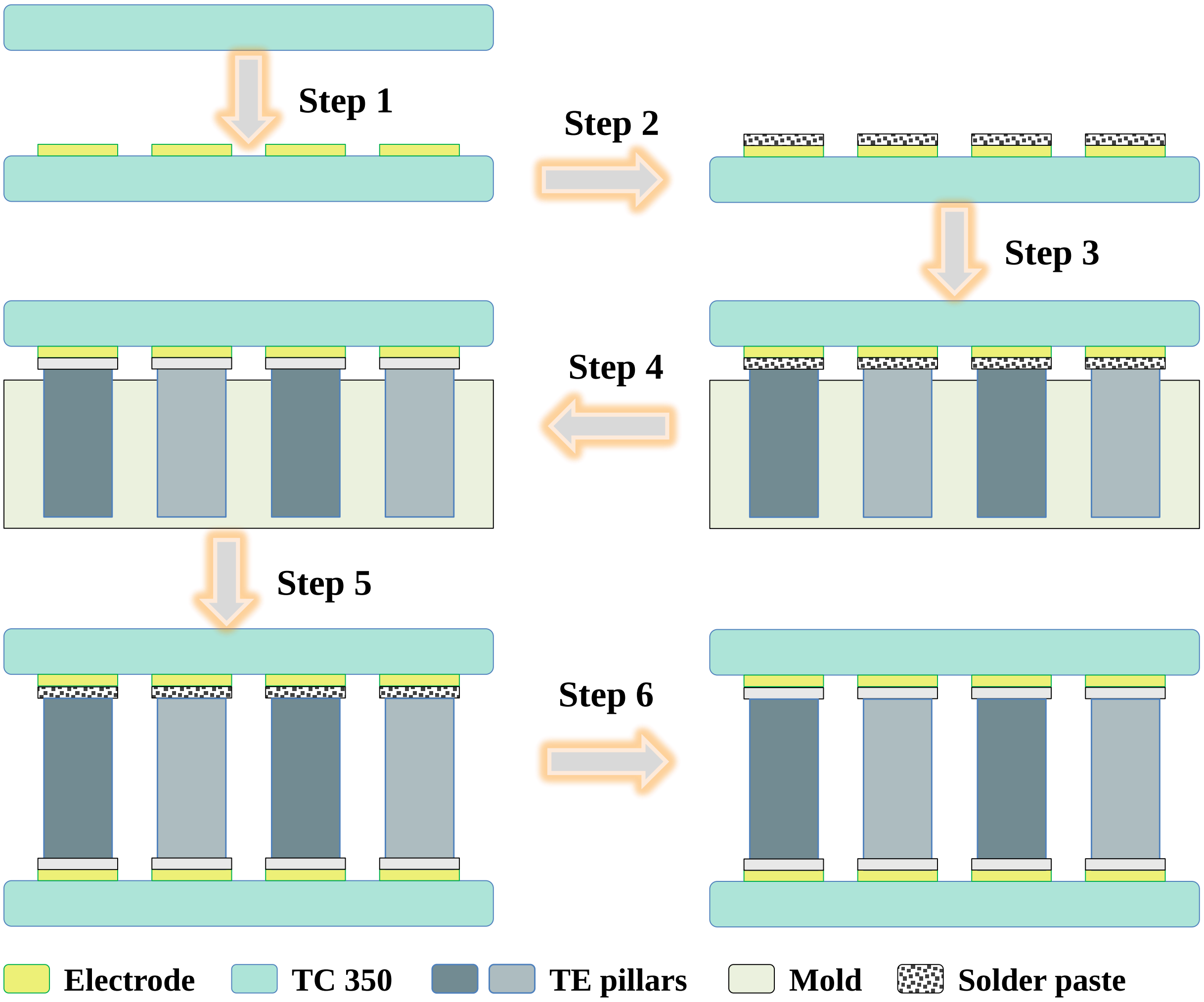

Figure S13. Fabrication process of our TEMD.
